# Assessing a Person-Centered and Culturally Sensitive Intervention for Arabic-, Turkish, or Urdu-Speaking Individuals With Type 2 Diabetes: Protocol for a Mixed Methods Realist Evaluation Study

**DOI:** 10.2196/69852

**Published:** 2025-09-11

**Authors:** Mie Klarskov Jensen, Natasja Bjerre

**Affiliations:** 1 Steno Diabetes Center Copenhagen Herlev Denmark

**Keywords:** immigrants, transients and migrants, minority groups, theoretical models, program evaluation, clinical protocols, public health, health personnel, artificial intelligence, AI, type 2 diabetes

## Abstract

**Background:**

Individuals from ethnic minority backgrounds have a 2.5 times higher incidence of type 2 diabetes (T2D) than ethnic Danes. They often face negative experiences with health care professionals, leading to unequal treatment. A person-centered and culturally adapted treatment approach can improve self-care, diabetes management, and treatment adherence.

**Objective:**

This mixed methods realist evaluation (RE) protocol aims to understand how a person-centered and culturally sensitive course of treatment for T2D works, for whom it is most effective, and under what circumstances it is likely to be effective.

**Methods:**

This RE is embedded within a 1-year randomized controlled trial, the Culturally Sensitive Course of Treatment for Individuals With T2D (ACCT2) study, for Arabic-, Turkish-, or Urdu-speaking individuals with suboptimal glycated hemoglobin (HbA_1c_ ; ≥53 mmol/mol) or unmet individual targets at 2 consecutive visits. The RE follows three phases: (1) developing, (2) testing, and (3) refining initial program theories. Data are collected through semistructured interviews at 4 months (visit 4) and at the end of the intervention (visit 6). Survey data are collected at baseline and at the end of the intervention. We aim to recruit 16 to 20 intervention participants, including at least 2 men and 2 women from each language group. Qualitative data will be analyzed thematically using a predefined codebook, and survey data will be analyzed descriptively.

**Results:**

As of December 2024, a total of 13 visits and 4 interviews had been completed. All baseline survey responses have been collected, along with 2 survey responses at the end of the intervention. Data analysis is pending. The results will inform revisions to the initial program theories, refining a comprehensive model that captures interactions between context, mechanisms, and outcomes. We anticipate disseminating the findings in the first half of 2026.

**Conclusions:**

This RE will support the randomized controlled trial by providing insights applicable to real-life clinical settings. The anticipated impact includes aiding the future development and implementation of interventions for the target groups.

**International Registered Report Identifier (IRRID):**

DERR1-10.2196/69852

## Introduction

### Background

Individuals with ethnic minority backgrounds constitute almost 16% of the Danish population, and this percentage is expected to increase [1]. Despite an improved focus on health among individuals with ethnic minority backgrounds, significant health inequity persists [2], and their incidence rate of type 2 diabetes (T2D) is 2.5 times greater that of ethnic Danes [3]. Individuals with ethnic minority backgrounds have an increased risk of developing severe T2D complications [3-6], partly due to genetic, environmental, cultural, and social factors challenging diabetes management [7]. The risk is further exacerbated by structural factors, as health information and services are often not adapted to the needs of ethnically diverse groups [8,9].

Previous research indicates that individuals with ethnic minority backgrounds often face negative experiences in consultations with health care professionals (HCPs), leading to mistrust and unequal treatment [10]. These challenges can stem from differing health perceptions [9,11], limited knowledge of the health care system [10,12], and reduced participation in diabetes educational interventions due to language barriers, lack of cultural sensitivity, and difficulties in navigating the health care system [13-15].

A person-centered and culturally adapted approach where HCPs pay attention to knowledge, motivation, individual experiences, values, cultural beliefs, and support from family and socioeconomic resources [16] has proven to support individuals with ethnic minority backgrounds in self-care and greater adherence to treatment plans [17]. This aligns with the realist evaluation (RE) framework proposed by Pawson and Tilley [18], which emphasizes understanding how context influences mechanisms that produce specific outcomes.

Previous studies have primarily focused on developing and testing group-based, culturally sensitive educational programs through randomized controlled trials (RCTs) and RE [19-22]. Although a previous study has developed a structured and person-centered model targeting the general diabetes population across both primary and secondary care sectors [23], there is a significant knowledge gap regarding how person-centered, culturally sensitive diabetes education and treatment work when integrated into clinical consultations for individuals with ethnic minority backgrounds in specialized diabetes care settings.

RCTs rarely address the context-specific factors that influence implementation outcomes or their connection to the underlying program theory [18], which limits the interpretability of findings across different programs and settings. Therefore, an RE is particularly valuable for assessing complex interventions in RCT studies, as it seeks to understand the generative causality between different intervention components and levels through the context-mechanism-outcome (CMO) configuration model, with a strong focus on context [18,24]. This approach enables a more holistic understanding of how and why an intervention works, moving beyond simple “what works” questions to explore “what works, for whom, in what circumstances, and why” [25].

Pawson and Tilley [18] acknowledge that interventions are active, adaptable to change, and embedded in a social reality that shapes both implementation and stakeholders’ responses to the intervention. This theoretical foundation is particularly relevant for understanding complex health care interventions targeting populations with ethnic minority backgrounds, where context plays a crucial role in determining effectiveness.

### Objectives

We have developed a 1-year, person-centered, and Culturally Sensitive Course of Treatment for Individuals With T2D (ACCT2 study [26]) to improve glycemic control (glycated hemoglobin; HbA_1c_), health literacy, and overall well-being. The target population includes men and women who speak Arabic, Turkish, or Urdu; have T2D; and have limited access to health care services. The overall purpose of this mixed methods RE protocol is to explore how the ACCT2 study works, for whom it is most effective, and under what circumstances it is likely to be effective. Specifically, we explore which intervention components contribute to specific outcomes by identifying the mechanisms that produce intended or unintended outcomes.

## Methods

### Study Design

Using a mixed methods design [27], we plan to conduct an RE [18] nested within the ACCT2 study [26]. The RE will follow the Realist and Meta-Narrative Evidence Synthesis: Evolving Standards guidelines [28] and will be reported separately. In the nested integration method, the RCT examines the causal impact of the ACCT2 study, while the RE provides a generative understanding of causality by explaining the intervention’s effectiveness through the interaction between mechanisms and context [29,30]. By developing and testing initial program theories of the intervention, we aim to produce robust evidence while maintaining high internal validity from the RCT, achieving both quantifiable outcomes and qualitative insights [31].

**Figure 1:**
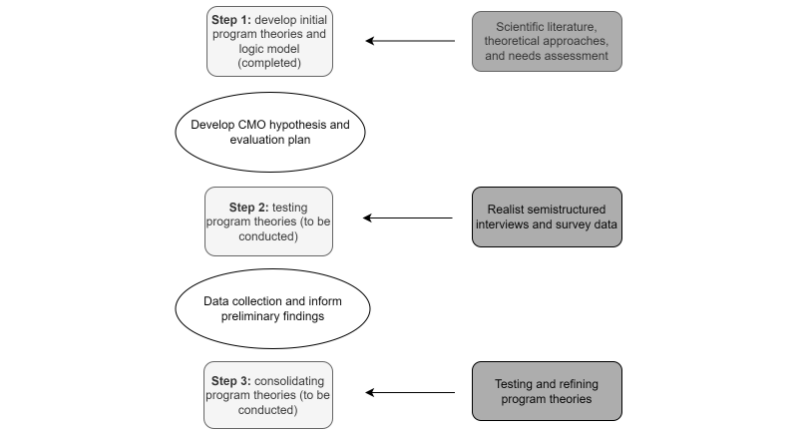
Study design and methods. CMO: context-mechanism-outcome.

In this RE, we understand mechanisms as “unobservable entities that are activated in a specific context to generate one or more outcomes” [30,32], meaning that the hidden mechanisms generate changes within the intervention. Through the RE, we use semistructured interviews and survey data to assess the effectiveness of the ACCT2 study thoroughly. The RE design involves three steps: (1) developing, (2) testing, and (3) consolidating our program theories based on data collection and analysis (Figure 1) [33].

**Figure 2:**
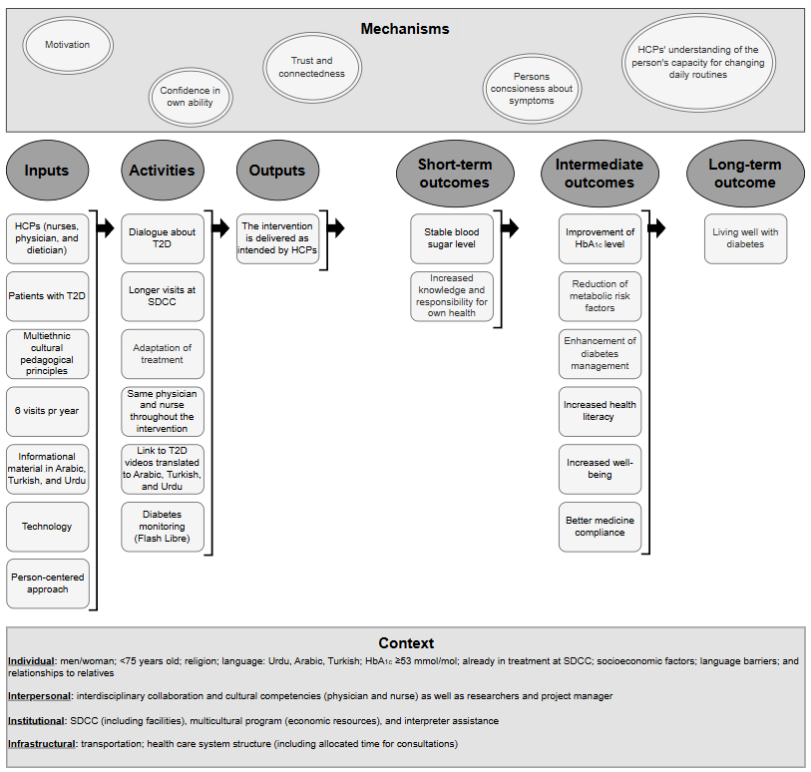
Initial logic model of a culturally sensitive course of treatment for individuals with type 2 diabetes, the Culturally Sensitive Course of Treatment for Individuals With T2D (ACCT2) study. HbA_1c_: glycated hemoglobin; HCP: health care professional; SDCC: Steno Diabetes Center Copenhagen; T2D: type 2 diabetes.

Our RE is guided by initial program theories [34] (step 1), outlining the mechanisms through which the ACCT2 study is expected to be effective within the context of Steno Diabetes Center Copenhagen (SDCC), Denmark. The initial program theories (Table 1) are depicted in a logic model (Figure 2) [18] following the CMO configuration framework and comprise resources; activities; outputs; and short-term, intermediate, and long-term outcomes [18]. The logic model [35] serves as a framework for organizing the theoretical and empirical assumptions underlying the ACCT2 study. It encompasses both the format and content of the designed intervention, and emphasizes that complex interventions possess underlying theories for achieving specific outcomes [36]. The initial program theories enable an examination of how the intervention works, for whom, and under what circumstances, focusing on the mechanisms it triggers and the resulting outcomes [36]. Applying a logic model and CMO configurations ensures a holistic and contextualized evaluation, integrating quantitative and qualitative data. Our initial program theories will guide our data collection and analysis in step 2.

In step 2, we plan to unfold and explore the mechanisms activated by the intervention by conducting semistructured interviews with participants, their close relatives, and HCPs delivering the intervention. We also plan to apply survey data collected during the RCT. By analyzing both qualitative and quantitative data, we will test the initial program theories to gain insights into the experiences and perspectives of participants.

In step 3, we will consolidate our initial program theories, based on our data analysis. Through the RE, we will focus on individual and interpersonal needs and institutional settings to design features that improve HbA_1c_ levels, health literacy, diabetes management, and well-being. This does not consider broader social and economic contexts, although it is based on the local context of SDCC. This study specifically captures behavioral, interpersonal, and social mechanisms that contribute to intervention outcomes.

**Table 1:** Initial program theories and rival theories of the culturally sensitive course of treatment for individuals with type 2 diabetes (ACCT2) study and evidence.

Theories		Evidence
**Consultations with the same HCP^a^**		
	Initial: ensuring longer and more consultations with the same HCPs will help build trusting relationships and dialogue. This will create a relaxed environment for the target group, allowing them to ask questions without feeling rushed. HCPs will understand and accommodate individual needs and treatment preferences better. This will lead to improved comprehension, adherence to treatment plans (eg, medication and healthy diet), and diabetes management, leading to better HbA_1c_^b^ levels and enhanced well-being.	[12]
	Rival: increasing the frequency and length of consultations will overwhelm the target group with excessive information, lowering retention and engagement, causing them to depend on future sessions to resolve issues, and weakening adherence to treatment plans. Overfamiliarity with the same HCPs can foster complacency rather than enhance communication. Consequently, HbA_1c_ levels and well-being will remain unchanged, and health care resources will be misallocated without improved outcomes.	[12]
**Increasing the number and duration of consultations**		
	Initial: expanding the number and duration of consultations (from 4 to 6 sessions, with an additional 30 min on average) will allow HCPs to thoroughly explain the nature of T2D^c^, complications, treatments, medications, and side effects. This will help the target group feel informed about their condition and have their questions addressed. This will increase their level of health literacy and encourage them to adhere to treatment plans (eg, medication, healthy diet, and physical activity) and diabetes management, leading to improved HbA_1c_ levels and enhanced well-being.	[12]
	Rival: expanding the number and duration of consultations might overwhelm the target group with information, leading to confusion rather than increased health literacy. Longer sessions could cause disengagement, with the target group burdened by the extra details. This can weaken their motivation to adhere to treatment plans, ultimately resulting in little to no improvement in HbA_1c_ levels or well-being. The extended consultations may also inefficiently use health care resources without significantly improving personal outcomes.	[12]
**Providing continuous glucose monitoring technology**		
	Initial: continuous glucose monitoring sensors will provide the target group with real-time blood glucose level data. These data will be discussed during consultations and used as pedagogic tools to empower and motivate the target group with concrete guidance on how to engage in diabetes management. As a result, the target group will be more likely to adhere to treatment plans (eg, medication, healthy diet, and physical activity), leading to improved HbA_1c_ levels and overall well-being.	[37-42]
	Rival: continuous glucose monitoring sensors will provide the target group with real-time blood glucose level data and increase their awareness of fluctuations. However, this heightened awareness may lead to greater anxiety and worry, as the target group becomes overfocused on data and guidance. This can result in poorer adherence to treatment plans and diabetes management, ultimately leading to poorer HbA_1c_ levels and overall well-being.	[37-42]
**Culturally adapted communication**		
	Initial: if HCPs use culturally adapted communication during consultations, such as translated information, tailored dialogue tools, and appropriate nonverbal methods when needed, the target group will better understand their condition. This will motivate them to act on the knowledge, as the materials will be accessible and culturally relevant. Consequently, this increased understanding will enhance health literacy and improve T2D management by aligning with cultural and linguistic backgrounds.	[43-46]
	Rival: if HCPs use culturally adapted communication with translated materials and tailored dialogue tools, the target group might not achieve better understanding or improved health literacy. Barriers such as personal beliefs, past experiences, or complex information can still impede comprehension and adherence, potentially limiting diabetes management.	[43-46]
**HCPs emphasize a holistic, whole-person perspective and diverse cultural context**		
	Initial: if HCPs have knowledge about diversity, cultures, and religions, then the target group will be more likely to engage in their treatment because the HCPs can plan and tailor the treatment to individual needs and diverse sociocultural contexts. This will result in HCPs perceiving the target group as more motivated to actively participate in their treatment because the customized treatment respects individual cultural and religious values and practices, which increases trust and willingness to cooperate.	[43,44,47]
	Rival: if HCPs have knowledge about diversity, cultures, and religions, the target group can still face barriers to fully engage in their treatment. While tailored care can help, other factors such as language barriers, socioeconomic challenges, or personal beliefs can continue to limit participation. Even with culturally sensitive treatment, the target group may prioritize traditional practices or remain hesitant to trust medical advice due to past experiences. As a result, it may not lead to increased trust, motivation, or willingness to cooperate.	[43,44,47]
**Relatives’ treatment responsibility**		
	Initial: if the target group receives individualized treatment and support from HCPs, then the relatives will no longer feel responsible for the treatment, because the target group will have gained the ability to manage their condition. This shift in responsibility occurs as the target group becomes more empowered to take charge of their health, reducing the burden on relatives.	[48,49]
	Rival: if the target group receives individualized treatment and support from HCPs, their relatives may still feel responsible for the treatment, as family involvement is often ingrained in caregiving roles. Despite gaining knowledge and the ability to manage T2D, relatives may continue to provide oversight or support out of concern or habit. This dynamic might persist, as relatives may not fully trust that the target group can manage T2D independently or may feel their emotional support is still necessary, thus maintaining a high level of responsibility.	[48,49]

^a^HCP: health care professional.

^b^HbA_1c_: glycated hemoglobin.

^c^T2D: type 2 diabetes.

### Participants

We aim to recruit a subgroup of 16 to 20 participants from the intervention group (N=48), with at least 2 men and 2 women from each language group to increase representativeness. The eligibility criteria for participants following the ACCT2 study are men and women who speak Arabic, Turkish, or Urdu as their native language; are already undergoing treatment at the outpatient T2D clinic at SDCC, Denmark; and have suboptimal HbA_1c_ levels (≥53 mmol/mol) or have not achieved their individual treatment goal for HbA_1c_ in 2 consecutive measurements [26]. Participants are stratified according to the 3 native languages included in the RCT (Arabic, Turkish, and Urdu). The final selection of participants for interviews is coordinated according to the timing of their visit 4 (V4). In addition, we plan to interview relatives from each language group (n=15) and HCPs delivering the intervention (physician: n=1 and nurse: n=1).

### Intervention Design and Key Components of the ACCT2 Study

The ACCT2 study was developed with the target group and HCPs from the outpatient T2D clinic at SDCC. It consists of three phases: (1) needs assessment, (2) intervention development and element testing, and (3) an RCT.

Phase 1 (needs assessment) involved interviews and workshops with individuals with T2D and non-Western backgrounds (n=18) and HCPs (nurses, physicians, and dieticians; n=18). The findings from this phase suggested adjustments to the standard course of treatment focusing on (1) extended duration and number of consultations, (2) use of technology for diabetes monitoring, (3) awareness of language barriers and use of culturally adapted communication, (4) continuity of care with the same HCPs, and (5) increasing levels of health literacy. A culturally sensitive approach by HCPs was considered vital for enhancing understanding and engagement in diabetes management among the target group, emphasizing a whole-person approach rooted in everyday life. On the basis of the needs assessment and evidence from existing literature, a 1-year intervention was designed and tested on a small scale in phase 2. Phase 3 involves a 1-year pragmatic RCT, including 96 individuals with T2D and an ethnic minority background. The ACCT2 study consists of 6 visits to SDCC (for further description, refer to the study protocol by Bjerre et al [26]). All visits are based on specific values and competencies of HCPs, which are important for the organization, implementation, and completion of the RCT. Educational materials, including written texts, images, and videos, have been translated into Arabic, Turkish, and Urdu. Beyond translation, the content has been culturally adapted to reflect participants’ values, food cultures, and visual preferences. Images feature individuals from the relevant ethnic backgrounds and illustrate familiar dietary practices to enhance cultural relevance and engagement [26].

To ensure and monitor intervention fidelity, detailed standard operating procedures were developed for both physician and nurse consultations. These standard operating procedures serve as fixed protocols that outline precisely how each component of the intervention should be delivered. Fidelity monitoring will be conducted through triangulation of data sources: medical records from the consultations are used to document the content and procedures followed, and the duration of each consultation is recorded as an indicator of adherence to the planned structure. In addition, the semistructured interviews included questions about participants’ experiences with key elements of the intervention to assess whether they perceived having received the intervention as intended. The CONSORT (Consolidated Standards of Reporting Trials) reporting guidelines were applied (Multimedia Appendix 1 [50]).

### Step 1: Developing Initial Program Theories of the ACCT2 Study

The initial program theories produce abstract and plausible explanatory insights into the components of the intervention and focus on how inputs and activities relate to specific responses of our target group (mechanisms), leading to a change in intended or observed outcomes [33,34,51]. Our initial program theories are based on scientific literature, theoretical approaches, and the needs assessment we conducted (Bjerre, N, unpublished data, July 2025). In Table 1, a list of initial program theories and rival theories is detailed and structured according to intervention components, that is, activities. The rival theories offer an alternative explanation that challenges the causal claims of program theories by suggesting other mechanisms that may simultaneously influence outcomes [24]. Rival theories, therefore, allow us to focus on the possible unintended intervention outcomes or harm [24].

### Step 2: Testing Program Theory

This step constitutes the core of the investigation, where the initial program theories from step 1 are tested through systematic data collection and analysis. On the basis of a data collection strategy (Table 2), it is specifically examined whether the expected relationships between context, mechanisms, and outcomes actually exist. A combination of quantitative and qualitative methods has been used to document both the occurrence of outcomes and the underlying processes that lead to them. In this process, it is central to investigate whether the theoretically expected mechanisms are activated in practice. This is examined through in-depth interviews to uncover the causal processes underlying the program’s effect or lack thereof. Simultaneously, it is systematically investigated how different contextual factors affect the program’s mode of operation. This may include demographic, organizational, geographic, or cultural differences that can explain variations in the intervention’s effectiveness across different settings. Throughout this process, attention will be paid to mechanisms or relationships that were not anticipated in the initial program theories. These findings are just as important as confirmation of existing theories and can lead to significant revisions in the understanding of how the intervention works [25].

**Table 2:** Initial data collection plan for the realist evaluation.

Constructs	Mode of operation	Data source	Participants	Measure	Time of data collection
Mechanisms	Motivation Empowerment Diabetes knowledge Capacity for change Trust and connectedness	Individual semistructured interviews	Persons (n=16), relatives (n=15), nurse (n=1), and physician (n=1)	Self-reported understanding of the meeting with health care professionals in the consultation	V4^a^, V6^b^, and every 6 months during the intervention^c^
Resources	Person-centered approach and cross-sectoral collaboration (culturally sensitive competencies)	Individual semistructured interviews	Persons (n=16), nurse (n=1), and physician (n=1)	Experience of change in behavior and the intervention process	V4, V6, and every 6 months during the intervention^c^
Activities	Dialogue of T2D^d^ (including diabetes technology) and adjustment of treatment	Individual semistructured interviews and survey data (REDCap^e^)	Persons (n=16), nurse (n=1), and physician (n=1)	Self-reported understanding of what has worked in the dialogue, and which factors have been most effective in adjusting the treatment	V4, V6, and every 6 months during the intervention^c^
Outputs	Intervention delivered as intended	Individual semistructured interviews and survey data (REDCap)	Persons (n=16), nurse (n=1), and physician (n=1)	Self-reported experience with access to diabetes knowledge, blood sugar measurement, and attendance to consultations	V4, V6, and every 6 months during the intervention^c^
Outcome	Increased knowledge and responsibility for own health	Individual semistructured interviews and survey data (REDCap)	Persons (n=16), relatives (n=15), nurse (n=1), and physician (n=1)	Individual experience of short-term and intermediate outcomes: health literacy, well-being, medication adherence, and diabetes management	V6 and every 6 months during the intervention^c^

^a^V4: visit 4.

^b^V6: visit 6.

^c^Data collected every 6 months during the intervention from nurse and physician.

^d^T2D: type 2 diabetes.

^e^REDCap: Research Electronic Data Capture (Vanderbilt University).

### Data Collection

Qualitative and survey data will be collected by the end of 2025.

#### Semistructured Interviews

To test the initial program theories (step 1), we will conduct semistructured interviews with participants at 2 time points during the RCT; following V4 and at the last visit (visit 6 [V6]). Relatives are invited to participate in one interview, while HCPs delivering the intervention will be interviewed every 6 months of the study period.

The RE interviews aim to explore and expand on various aspects of the program theories. Different interviewguides will be constructed for the target group, relatives, and HCPs to explore experiences during the intervention, assess its acceptability, and identify potential improvements and key factors influencing the effectiveness of the intervention. Before the participants’ V4 time point, we will call to schedule the interview to take place immediately after their V4 appointment, during which we will meet them. Before conducting interviews, participants will receive oral information about the interview, and written informed consent will be obtained.

Qualitative researchers will conduct the interviews at SDCC, lasting 20 to 30 minutes.

#### Survey Data

Quantitative data, in the form of survey data collected using validated questionnaires (for further details, refer to RCT protocol by Bjerre et al [26]), will be gathered at baseline visit 1 and after the end of the intervention (V6; Table 3). The survey data cover well-being, diabetes management, health literacy, medication adherence, and satisfaction with the intervention. Participants can complete the survey in Danish, Arabic, Turkish, or Urdu.

**Table 3:** Questionnaires used in the survey at visit 1 and visit 6.

Questionnaires	Baseline (visit 1)	End (visit 6)
Sociodemographic characteristics	✓	
Diabetes distress (PAID-5^a^) [52]	✓	✓
Well-being (WHO-5^b^) [53]	✓	✓
HLQ^c^ (3 items) [54]	✓	✓
Diabetes management (PRO^d^ scheme diabetes) [55]	✓	✓
MARS-5^e^ [56]	✓	✓
Acceptability, retention, and satisfaction [57]	✓	✓

^a^PAID-5: Problem Areas in Diabetes Scale–5.

^b^WHO-5: World Health Organization–Five Well-Being Index.

^c^HLQ: Health Literacy Questionnaire.

^d^PRO: patient-reported outcome.

^e^MARS: Medication Adherence Rating Scale.

### Data Analysis

The data analysis will be guided by the initial program theories, which form the foundation for the codebook comprising conceptual categories related to the CMO configurations. The audio records from the interviews will be transcribed verbatim using the artificial intelligence software Viceron27 (Centre for IT and Medical Technology) and subsequently reviewed by qualitative researchers to ensure accuracy. Two researchers will conduct a deductive thematic analysis based on a predefined codebook, with a third researcher consulted in cases of disagreement.

Meaningful text units will be coded in Word (Microsoft Corp) according to the codebook and subsequently organized in Excel (Microsoft Corp) spreadsheets, structured by each initial program theory. CMO components will be extracted from the coded transcripts, accompanied by analytic notes and reflections to explore how context and mechanisms interact to generate specific outcomes.

On the basis of these analyses, revised CMO configurations will be developed to describe how particular mechanisms are triggered within given contextual conditions. These revised CMO configurations will then be compared to the initial CMO configurations based on the initial program theories to assess which patterns were confirmed, refined, or refuted. Particular attention will be paid to the relationships between the following dyads: context-outcome, mechanism-outcome, and context- mechanism dyads.

In parallel, survey data will be analyzed descriptively as part of the RCT [26], with relevant findings integrated into the RE to elaborate on the initial program theories. The statistical analysis is described in detail in the RCT protocol [26].

### Step 3: Consolidating Program Theories

In this phase, we will refine and update our initial program theories using the qualitative and quantitative insights from the RE data analysis in step 2 to ensure a robust model that accurately captures the complex relationships between context, mechanisms, and outcomes of the ACTT2 study. This involves refining existing CMO configurations, removing theories not supported by data, and developing new theories based on unexpected findings. Across different datasets and contexts, patterns are identified to develop a broader understanding of which factors consistently affect the intervention’s impact. This may reveal underlying principles or “meta-mechanisms” that operate across different specific contexts. Simultaneously, the focus will also be on context specification, with a description of the conditions under which the intervention functions optimally, moderately, or not at all. This often involves identifying configurations of contextual factors that are necessary to activate positive mechanisms [25].

The consolidated CMO configurations are then adapted into the initial program theories. The revised program theories include both strategies for optimizing existing contexts and recommendations for adapting the intervention to different contextual conditions. Finally, the developed theories are positioned in relation to existing research literature and identify how they contribute to the broader understanding of similar interventions and phenomena [25].

### Patient and Public Involvement

The intervention was developed in close collaboration with the target group and HCPs, using interviews and workshops (n=18 for each), ensuring that their perspectives and needs were integrated into the research and design process. This active involvement helped highlight key areas for improving health care delivery and emphasized the need for a culturally sensitive approach to enhance engagement and diabetes management. In addition, participants’ perspectives are thoroughly considered throughout the evaluation, including potential burdens of the intervention and time requirements. All participants will be invited to seminars where we will present overall findings, allowing them to highlight which results are most critical to share with the SDCC outpatient clinic and the broader public. The participants are covered by the Patient Compensation Association under the Danish Act on the Right to Complain and Receive Compensation in the Health Service.

### Ethical Considerations

The study was approved by the Capital Region of Denmark Ethics Committee (H-387 23042245) and will be conducted in line with the Declaration of Helsinki. It complies with the Danish Data Protection Agency and the General Data Protection Regulation. We will secure adequate blinding of personal data during processing and publication. Participants are covered by the Person Compensation Association. All participants will sign a written informed consent. They will be informed about their right to withdraw at any time and will be guaranteed confidentiality.

## Results

As of December 2024, a total of 13 participants have been enrolled, with 13 V4 interviews completed. All baseline survey responses have been collected, along with 2 survey responses at the end of the intervention. Data analysis is pending. The results will inform revisions to the initial program theories, refining a comprehensive model that accurately captures the interactions between context, mechanisms, and outcomes in the ACCT2 study. The study findings will be disseminated in peer-reviewed publications and conference presentations in 2026 or 2027.

## Discussion

### Anticipated Findings

This protocol presents the design of an RE of the ACCT2 study, aiming to investigate which intervention components contribute to specific outcomes by identifying the mechanisms that produce intended or unintended outcomes. Furthermore, it seeks to understand how the intervention works, for whom it is most effective, and under what circumstances it is likely to be effective.

To our knowledge, this will be the first comprehensive RE to explore how culturally sensitive, person-centered diabetes treatment functions within outpatient consultations for individuals with T2D who speak Arabic, Turkish, or Urdu. We expect that our refined program theory, based on the study findings, will be supported by previous results from other REs and by existing theoretical and empirical evidence in the field.

### Strengths and Limitations

A methodological strength of the design is that, before performing this study, the intervention was developed based on a needs assessment involving the target group and HCPs, ensuring an in-depth understanding of their needs, barriers, and potential solutions. In addition, including participants of both genders and from diverse language groups (Arabic, Turkish, and Urdu) will enable us to explore variations in the intervention’s effectiveness across these demographics.

To enhance the theoretical rigor of our approach, a subject matter expert in RE supported us in reviewing and refining the initial program theories to ensure that they align with the ACCT2 study’s framework.

A limitation of the RE design is its lack of generalizability, largely due to the significant influence of the specific context of the intervention. In addition, potential language barriers among participants are expected and may result in some information being withheld or explanations being shortened.

The target group is known to be grateful and respectful of authority and may hesitate to share negative experiences, which could affect the validity of the data. To address this, participants were encouraged to share both positive and critical feedback to help improve the intervention. Since both interviewers may have interacted with participants during recruitment and data collection, there is a risk of interviewer bias due to power asymmetry and social desirability. This is mitigated by emphasizing openness, triangulating with questionnaires and existing evidence, and encouraging critical reflections. The project manager, who contributed to the development of initial program theories and interview guides due to in-depth knowledge of the intervention, was kept out of data collection and analysis to reduce potential bias. Nonetheless, confirmation bias remains a risk, as the interview guide is shaped by early theories and may overlook alternative explanations, particularly when language barriers are present.

### Conclusions

Our RE will elucidate the program theories of the ACCT2 study and address the knowledge gaps highlighted by these theories. This RE is expected to support the RCT by offering insights applicable to real-life clinical settings. The anticipated impact includes aiding the future development and implementation of interventions for individuals with ethnic minority backgrounds and T2D and to minimize inequality in health and T2D treatment.

## Appendix

Multimedia Appendix 1 CONSORT 2025 editable checklist [38].

## Abbreviations

ACCT2: Culturally Sensitive Course of Treatment for Individuals With T2D
CMO: context-mechanism-outcome
HbA_1c_: glycated hemoglobin
HCP: health care professional
RCT: randomized controlled trial
RE: realist evaluation
SDCC: Steno Diabetes Center Copenhagen
T2D: type 2 diabetes
V4: visit 4
V6: visit 6
