# Supplementary material for: Assessing a Person-Centered and Culturally Sensitive Intervention for Arabic-, Turkish, or Urdu-Speaking Individuals With Type 2 Diabetes: Protocol for a Mixed Methods Realist Evaluation Study
Source: JMIR Res Protoc. 2025 Sep 11;14:e69852. doi: 10.2196/69852 (PMC12464501; doi:10.2196/69852)
Supplement: Multimedia Appendix 1 [file resprot_v14i1e69852_app1.docx]

|  | Section/topic | No | CONSORT 2025 checklist item description | Reported on page no. |
| --- | --- | --- | --- | --- |
|  | **Title and abstract** | | |  |
|  | Title and structured abstract | 1a | Identification as a randomised trial | 1 |
|  |  | 1b | Structured summary of the trial design, methods, results, and conclusions | 2 |
|  | **Open science** | | |  |
|  | Trial registration | 2 | Name of trial registry, identifying number (with URL) and date of registration | 3 |
|  | Protocol and statistical analysis plan | 3 | Where the trial protocol and statistical analysis plan can be accessed | DOI: [10.2196/67319](https://doi.org/10.2196/67319) Multimedia Appendix 1  CONSORT-eHEALTH checklist (V 1.6.1) |
|  | Data sharing | 4 | Where and how the individual de-identified participant data (including data dictionary), statistical code and any other materials can be accessed | DOI: [10.2196/67319](https://doi.org/10.2196/67319) Multimedia Appendix 1  CONSORT-eHEALTH checklist (V 1.6.1) |
|  | Funding and conflicts of interest | 5a | Sources of funding and other support (eg, supply of drugs), and role of funders in the design, conduct, analysis and reporting of the trial | 21 |
|  |  | 5b | Financial and other conflicts of interest of the manuscript authors | 21 |
|  | **Introduction** | | |  |
|  | Background and rationale | 6 | Scientific background and rationale | 3-5 |
|  | Objectives | 7 | Specific objectives related to benefits and harms | 5 |
|  | **Methods** | | |  |
|  | Patient and public involvement | 8 | Details of patient or public involvement in the design, conduct and reporting of the trial | 17-18 DOI: [10.2196/67319](https://doi.org/10.2196/67319) Multimedia Appendix 1  CONSORT-eHEALTH checklist (V 1.6.1) |
|  | Trial design | 9 | Description of trial design including type of trial (eg, parallel group, crossover), allocation ratio, and framework (eg, superiority, equivalence, non-inferiority, exploratory) | 8-9 DOI: [10.2196/67319](https://doi.org/10.2196/67319) Multimedia Appendix 1  CONSORT-eHEALTH checklist (V 1.6.1) |
|  | Changes to trial protocol | 10 | Important changes to the trial after it commenced including any outcomes or analyses that were not prespecified, with reason | DOI: [10.2196/67319](https://doi.org/10.2196/67319) Multimedia Appendix 1  CONSORT-eHEALTH checklist (V 1.6.1) |
|  | Trial setting | 11 | Settings (eg, community, hospital) and locations (eg, countries, sites) where the trial was conducted | 8 DOI: [10.2196/67319](https://doi.org/10.2196/67319) Multimedia Appendix 1  CONSORT-eHEALTH checklist (V 1.6.1) |
|  | Eligibility criteria | 12a | Eligibility criteria for participants | 7 DOI: [10.2196/67319](https://doi.org/10.2196/67319) Multimedia Appendix 1  CONSORT-eHEALTH checklist (V 1.6.1) |
|  |  | 12b | If applicable, eligibility criteria for sites and for individuals delivering the interventions (eg, surgeons, physiotherapists) | Not applicable |
|  | Intervention and comparator | 13 | Intervention and comparator with sufficient details to allow replication. If relevant, where additional materials describing the intervention and comparator (eg, intervention manual) can be accessed | DOI: [10.2196/67319](https://doi.org/10.2196/67319) Multimedia Appendix 1  CONSORT-eHEALTH checklist (V 1.6.1) |
|  | Outcomes | 14 | Prespecified primary and secondary outcomes, including the specific measurement variable (eg, systolic blood pressure), analysis metric (eg, change from baseline, final value, time to event), method of aggregation (eg, median, proportion), and time point for each outcome | DOI: [10.2196/67319](https://doi.org/10.2196/67319) Multimedia Appendix 1  CONSORT-eHEALTH checklist (V 1.6.1) |
|  | Harms | 15 | How harms were defined and assessed (eg, systematically, non-systematically) | DOI: [10.2196/67319](https://doi.org/10.2196/67319) Multimedia Appendix 1  CONSORT-eHEALTH checklist (V 1.6.1) |
|  | Sample size | 16a | How sample size was determined, including all assumptions supporting the sample size calculation | DOI: [10.2196/67319](https://doi.org/10.2196/67319) Multimedia Appendix 1  CONSORT-eHEALTH checklist (V 1.6.1) |
|  |  | 16b | Explanation of any interim analyses and stopping guidelines | DOI: [10.2196/67319](https://doi.org/10.2196/67319) Multimedia Appendix 1  CONSORT-eHEALTH checklist (V 1.6.1) |
|  | Randomisation: |  |  |  |
|  | Sequence generation | 17a | Who generated the random allocation sequence and the method used | DOI: [10.2196/67319](https://doi.org/10.2196/67319) Multimedia Appendix 1  CONSORT-eHEALTH checklist (V 1.6.1) |
|  |  | 17b | Type of randomisation and details of any restriction (eg, stratification, blocking and block size) | DOI: [10.2196/67319](https://doi.org/10.2196/67319) Multimedia Appendix 1  CONSORT-eHEALTH checklist (V 1.6.1) |
|  |  |  |  | **Reported on page no.** |
|  | Allocation concealment mechanism | 18 | Mechanism used to implement the random allocation sequence (eg, central computer/telephone; sequentially numbered, opaque, sealed containers), describing any steps to conceal the sequence until interventions were assigned | DOI: [10.2196/67319](https://doi.org/10.2196/67319) Multimedia Appendix 1  CONSORT-eHEALTH checklist (V 1.6.1) |
|  | Implementation | 19 | Whether the personnel who enrolled and those who assigned participants to the interventions had access to the random allocation sequence | DOI: [10.2196/67319](https://doi.org/10.2196/67319) Multimedia Appendix 1  CONSORT-eHEALTH checklist (V 1.6.1) |
|  | Blinding | 20a | Who was blinded after assignment to interventions (eg, participants, care providers, outcome assessors, data analysts) | DOI: [10.2196/67319](https://doi.org/10.2196/67319) Multimedia Appendix 1  CONSORT-eHEALTH checklist (V 1.6.1) |
|  |  | 20b | If blinded, how blinding was achieved and description of the similarity of interventions | DOI: [10.2196/67319](https://doi.org/10.2196/67319) Multimedia Appendix 1  CONSORT-eHEALTH checklist (V 1.6.1) |
|  | Statistical methods | 21a | Statistical methods used to compare groups for primary and secondary outcomes, including harms | DOI: [10.2196/67319](https://doi.org/10.2196/67319) Multimedia Appendix 1  CONSORT-eHEALTH checklist (V 1.6.1) |
|  |  | 21b | Definition of who is included in each analysis (eg, all randomised participants), and in which group | DOI: [10.2196/67319](https://doi.org/10.2196/67319) Multimedia Appendix 1  CONSORT-eHEALTH checklist (V 1.6.1) |
|  |  | 21c | How missing data were handled in the analysis | DOI: [10.2196/67319](https://doi.org/10.2196/67319) Multimedia Appendix 1  CONSORT-eHEALTH checklist (V 1.6.1) |
|  |  | 21d | Methods for any additional analyses (eg, subgroup and sensitivity analyses), distinguishing prespecified from post hoc | DOI: [10.2196/67319](https://doi.org/10.2196/67319) Multimedia Appendix 1  CONSORT-eHEALTH checklist (V 1.6.1) |
|  | **Results** | | |  |
|  | Participant flow, including flow diagram | 22a | For each group, the numbers of participants who were randomly assigned, received intended intervention, and were analysed for the primary outcome | DOI: [10.2196/67319](https://doi.org/10.2196/67319) Multimedia Appendix 1  CONSORT-eHEALTH checklist (V 1.6.1) |
|  |  | 22b | For each group, losses and exclusions after randomisation, together with reasons | DOI: [10.2196/67319](https://doi.org/10.2196/67319) Multimedia Appendix 1  CONSORT-eHEALTH checklist (V 1.6.1) |
|  | Recruitment | 23a | Dates defining the periods of recruitment and follow-up for outcomes of benefits and harms | DOI: [10.2196/67319](https://doi.org/10.2196/67319) Multimedia Appendix 1  CONSORT-eHEALTH checklist (V 1.6.1) |
|  |  | 23b | If relevant, why the trial ended or was stopped | Not relevant |
|  | Intervention and comparator delivery | 24a | Intervention and comparator as they were actually administered (eg, where appropriate, who delivered the intervention/comparator, how participants adhered, whether they were delivered as intended (fidelity)) | DOI: [10.2196/67319](https://doi.org/10.2196/67319) Multimedia Appendix 1  CONSORT-eHEALTH checklist (V 1.6.1) |
|  |  | 24b | Concomitant care received during the trial for each group | DOI: [10.2196/67319](https://doi.org/10.2196/67319) Multimedia Appendix 1  CONSORT-eHEALTH checklist (V 1.6.1) |
|  | Baseline data | 25 | A table showing baseline demographic and clinical characteristics for each group | DOI: [10.2196/67319](https://doi.org/10.2196/67319) Multimedia Appendix 1  CONSORT-eHEALTH checklist (V 1.6.1) |
|  | Numbers analysed,  outcomes and estimation | 26 | For each primary and secondary outcome, by group:  ● the number of participants included in the analysis  ● the number of participants with available data at the outcome time point  ● result for each group, and the estimated effect size and its precision (such as 95% confidence interval)  ● for binary outcomes, presentation of both absolute and relative effect size | DOI: [10.2196/67319](https://doi.org/10.2196/67319) Multimedia Appendix 1  CONSORT-eHEALTH checklist (V 1.6.1) |
|  | Harms | 27 | All harms or unintended events in each group | DOI: [10.2196/67319](https://doi.org/10.2196/67319) Multimedia Appendix 1  CONSORT-eHEALTH checklist (V 1.6.1) |
|  | Ancillary analyses | 28 | Any other analyses performed, including subgroup and sensitivity analyses, distinguishing pre-specified from post hoc | DOI: [10.2196/67319](https://doi.org/10.2196/67319) Multimedia Appendix 1  CONSORT-eHEALTH checklist (V 1.6.1) |
|  | **Discussion** | | |  |
|  | Interpretation | 29 | Interpretation consistent with results, balancing benefits and harms, and considering other relevant evidence | DOI: [10.2196/67319](https://doi.org/10.2196/67319) Multimedia Appendix 1  CONSORT-eHEALTH checklist (V 1.6.1) |
|  | Limitations | 30 | Trial limitations, addressing sources of potential bias, imprecision, generalisability, and, if relevant, multiplicity of analyses | DOI: [10.2196/67319](https://doi.org/10.2196/67319) Multimedia Appendix 1  CONSORT-eHEALTH checklist (V 1.6.1) |

Citation: Hopewell S, Chan AW, Collins GS, Hróbjartsson A, Moher D, Schulz KF, et al. CONSORT 2025 Statement: updated guideline for reporting randomised trials. BMJ. 2025; 388:e081123. <https://dx.doi.org/10.1136/bmj-2024-081123>
© 2025 Hopewell et al. This is an Open Access article distributed under the terms of the Creative Commons Attribution License (<https://creativecommons.org/licenses/by/4.0/>), which permits unrestricted use, distribution, and reproduction in any medium, provided the original work is properly cited.

*We strongly recommend reading this statement in conjunction with the CONSORT 2025 Explanation and Elaboration and/or the CONSORT 2025 Expanded Checklist for important clarifications on all the items. We also recommend reading relevant CONSORT extensions. See [www.consort-spirit.org](http://www.consort-spirit.org).
